# Concord Grape Juice Polyphenols and Cardiovascular Risk Factors: Dose-Response Relationships

**DOI:** 10.3390/nu7125519

**Published:** 2015-12-02

**Authors:** Jeffrey B. Blumberg, Joseph A. Vita, C. -Y. Oliver Chen

**Affiliations:** 1 Antioxidants Research Laboratory, Jean Mayer USDA Human Nutrition Research Center on Aging Tufts University, Boston, MA 02111, USA; 2 Evans Department of Medicine and the Whitaker Cardiovascular Institute, Boston University School of Medicine, Boston, MA 02118, USA; jvita@bu.edu; 3 Antioxidants Research Laboratory, Jean Mayer USDA Human Nutrition Research Center on Aging Tufts University, Boston, MA 02111, USA; oliver.chen@tufts.edu

**Keywords:** concord grape juice, polyphenols, flavonoids, cardiovascular risk factors, blood pressure, platelet aggregation, flow-mediated dilation, LDL oxidation

## Abstract

Pure fruit juices provide nutritional value with evidence suggesting some of their benefits on biomarkers of cardiovascular disease risk may be derived from their constituent polyphenols, particularly flavonoids. However, few data from clinical trials are available on the dose-response relationship of fruit juice flavonoids to these outcomes. Utilizing the results of clinical trials testing single doses, we have analyzed data from studies of 100% Concord grape juice by placing its flavonoid content in the context of results from randomized clinical trials of other polyphenol-rich foods and beverages describing the same outcomes but covering a broader range of intake. We selected established biomarkers determined by similar methods for measuring flow-mediated vasodilation (FMD), blood pressure, platelet aggregation, and the resistance of low density lipoprotein cholesterol (LDL) to oxidation. Despite differences among the clinical trials in the treatment, subjects, and duration, correlations were observed between the dose and FMD. Inverse dose-response relationships, albeit with lower correlation coefficients, were also noted for the other outcomes. These results suggest a clear relationship between consumption of even modest serving sizes of Concord grape juice, flavonoid intake, and effects on risk factors for cardiovascular disease. This approach to dose-response relationships may prove useful for testing other individual foods and beverages.

## 1. Introduction

The consumption of 100% fruit juice is positively associated with improved diet quality in children and adults [1] and with essential nutrient adequacy in children, and thus can contribute to a healthy diet [2]. Further, the phytochemical content of pure fruit juices, especially their complex profile of polyphenols, may contribute importantly to several of the functional benefits associated with their consumption. Indeed, in discussing the preponderance of evidence regarding the nutritional value of 100% fruit juice and its role in helping people meet recommendations for fruit intake, Clemens *et al.* [3] note the importance of fruit juice in delivering not only essential nutrients but also polyphenols. An extensive body of scientific literature supports a beneficial role for dietary polyphenols, particularly the flavonoids, in reducing the risk for cardiovascular disease (CVD). However, absent a framework like the Dietary Reference Intakes for essential nutrients, Lupton *et al.* [4] have recently described an evaluative process for substantiating the relationship between dietary bioactive components such as the polyphenols and enhanced health outcomes or reduced risk of chronic disease. Criteria defined for this process include validated methods of analysis of the bioactives, databases quantifying the presence of these compounds in diets, human studies characterizing their pharmacokinetic profiles, and a biological plausibility for their efficacy in promoting health. Providing a plausible biological explanation for the efficacy of dietary polyphenols is a large body of evidence (e.g., see Del Rio *et al.* [5]) with information available as well on some specific foods such as Concord grape juice. For example, Concord and other purple grape juice flavonoids have been demonstrated to increase antioxidant defenses, reduce inflammation, promote vascular reactivity, and inhibit atherogenesis [6,7]. However, most of the available relevant information on flavonoid bioavailability, distribution, metabolism, and excretion has been derived from studies of just one grape variety, Concord grape juice [8,9,10]. Thus, we utilized the clinical data from the Concord grape juice studies to assess the dose-relationship between cardiovascular disease risk and purple grape juice polyphenols. 

Lupton *et al.* [4] also list as important evaluative criteria information from prospective cohort studies and clinical trials on efficacy and dose-response data. There are several prospective cohort studies investigating the relationship between flavonoids and health outcomes but few of which examine closely such intake from individual food items. There are also a limited number of randomized clinical trials directed to research on efficacy regarding specific outcomes from a specific food intervention. Regrettably, little or no data on dose-response relationships are available from most clinical trials. The diversity of analytical methods for determining the polyphenol content of the test food and limitation of most studies to only one or two CVD risk factors prevent the application of a robust systematic review of this topic. However, there is an opportunity to explore the available data from these research studies to learn more about the effect of a specific food, here 100% Concord grape juice, and extract information useful for generating dose-response data applicable to elucidating information about serving sizes effective for health outcomes. 

## 2. Background: Polyphenol Content of Grape Juices and Their Contribution to Dietary Intakes

### 2.1. Polyphenol Content and Profile of Grape Juice

The polyphenol content and profile of grape juice is substantially dependent upon the variety from which it is prepared, though environmental factors and methods of cultivation can affect both. Grapes are typically rich in anthocyanins, flavanols (or flavan-3-ols or catechins), flavonols, proanthocyanidins, and stilbenes (Table 1). However, regrettably, some of the literature does not provide critical information about the grape variety (or combination of varieties) being investigated. Mullen *et al.* [11] characterized by HPLC-PDA, HPLC-MS^2^, and thiolysis assays the phenolic compounds in Welch’s Purple Grape juice made from Concord grapes and Tesco Pure Pressed Red Grape juice and reported their total content in µmol/L as 968 ± 11 (*n* = 26 identified phenolic compounds) and 92 ± 1 (*n* = 16 identified phenolic compounds), respectively; however, these numbers of phenolic compounds are underestimated as they do not reflect the flavanols determined by thiolysis which account for 434 and 10 µmol/L of the total content, respectively. The USDA Database for the Flavonoid Content of Selected Foods [12] lists 100% grape juice (canned or bottled, unsweetened, without vitamin C) as containing per 100 mL: 17.08 mg anthocyanidins, 1.47 mg flavanols, 0.02 mg flavones, and 1.52 mg flavonols; white grape juice as containing per 100 mL: 0.18 mg flavanols and 0.10 mg flavonols; and black grape juice as containing 0.85 mg flavanols/100 mL. Gu *et al.* [13] determined the concentration of total proanthocyanidins (including monomers through >10 mers) by HPLC-MS^2^ in grape juice as 52.4 ± 2 mg/100 mL. The USDA Database for the Proanthocyanidin Content of Selected Foods [14] lists purple grape juice as containing 52.03 mg/100 mL total proanthocyanidins (monomers through polymers), including prodelphinidins. In Table 1, among the anthocyanins (glucosides of anthocyanidins), Mullen *et al.* [11] characterized 21 compounds in the Concord grape juice and nine in red grape juice, including two and three phenolic acids, respectively. Three and four flavonols were identified in the Concord and red grape juice, respectively. Stalmach *et al.* [8] quantified 25 anthocyanins, seven phenolic acids, five flavanols, 10 flavonols, and resveratrol in the Concord grape juice. Similarly, Wang *et al.* [15] identified 27 anthocyanins in Concord grape juice using HPLC/DAD-MS. 

There is substantial overlap in the composition of Concord grape juice with that of red grape juice with regard to their flavonoid profiles. For example, of the nine anthocyanins found in red grape juice, only one is not present in Concord grape juice; conversely, of the twenty one anthocyanins found in Concord grape juice, 13 are not present in red grape juice [11]. Of the three flavonols identified in Concord grape juice, all are found among the four present in red grape juice [11]. In considering the benefits of long-term consumption of fruit juices, Mullen *et al.* [11] suggested that their “protective effects may be enhanced by consumption of a combination of juices rich in phenolics and containing a diverse variety of individual phenolic compounds.” The hypothesis that combining the flavonoid profiles from different grapes and/or other berries within a fruit juice could yield additive or synergistic effects with regard to their bioavailability or bioactivity is reasonable but requires substantiating evidence (e.g., see Borges *et al.* [16]).

In addition to its phenolic constituents, it is worthwhile noting that some 100% grape juices may be fortified with vitamin C which has been shown to act in synergy with flavonoids to increase the resistance of LDL to oxidation and may contribute to some of the outcomes achieved in clinical studies [17].

### 2.2. Dietary Intake of Flavonoids

Considering grape juice in the context of dietary patterns can help to determine its contribution to the total daily intake of flavonoids and their association with the risk of CVD. Utilizing the NHANES 1999–2002 database, Chun *et al.* [18] reported the daily flavonoid intake of U.S. adults ≥19 years as 3.1 ± 0.5 mg anthocyanidins, 156.5 ± 11.3 mg flavanols, and 12.9 mg flavonols. Gu *et al.* [13] estimated the mean daily intake of proanthocyanidins for all Americans >2 years at 57.7 mg/day and for men and women 40–59 years, at 64.6 and 56.7 mg/day, respectively. Based on the average values listed in Table 1, a 118 and 237 mL (~4 and 8 fl oz) serving of Concord grape juice, which is equivalent to a half cup and one cup of fruit according to the USDA dietary guidelines [19], provides an average of 14.73 to 29.58 mg proanthocyanidins, 31.69 to 63.65 mg anthocyanins, 1.65 to 3.32 mg flavanols, and 4.28 to 8.59 mg flavonols or about 52.34 to 105.13 mg total flavonoids in 306.80 to 616.20 mg of total phenolic compounds. Thus, flavonoids from even a modest serving of Concord grape juice can contribute substantially to the total daily intake of polyphenols. While the pharmacokinetic profile of grape juice polyphenols is outside the scope of this report, it is worth noting that the absorption and metabolism of flavonoids and phenolic acids from 100% Concord grape juice are similar to that of other berry fruit juices. For example, the parent compounds from Concord grape juice were found in low concentrations in plasma and urine with about 40 metabolites present in plasma and urine within 24 h, principally as glucuronide, methyl, and/or sulfate conjugates of anthocyanins and flavanols [8]. Peak plasma concentrations in healthy subjects ranged from 1.0 nmol/L for petunidin-3-*O*-glucoside to 355 nmol/L for dihydrocoumaric acid [9]. Urinary excretion, an indicator of bioavailability, varied from 0.26% for total anthocyanins to 24% for metabolites of hydroxycinnamate tartarate esters [9]. Studies in healthy people and ileostomists indicate that grape juice flavonoids and phenolic acids pass from the small intestine to the colon where an array of aromatic compounds are produced by gut microbiota; these metabolites are more predominant than phase I and II metabolites originating from absorption in the upper gastrointestinal tract [10].

**Table 1:** Phenolic Compounds in Grape Varieties *.

Phenolic Class	Purple ^1,2^	Black ^1^	Red ^1^	White ^1,2^	White ^3^	Concord Purple ^4^	Red ^4^	Bordo Purple ^5^	Concord Purple ^6^	Concord Purple ^7^	Purple ^8^ (Variety Not Specified)	Bordo Purple ^9^	Average Concord	Average Purple ^c^	Average White
	mg/100 mL														
Flavanols	1.47	0.85	0	0.18	1.47			2.42	2.8				1.4	2.23	0.82
Anthocyanins	17.08		0.52			15.41	1.51	10.87	38.3				26.86	20.42	
Flavones	0.02		0										0	0.02	
Flavonols	1.52		0.73	0.10	0.05	3.65	0.84		3.6				3.63	2.92	0.07
Proanthocyanidins	52.03 ^b^			0.42	1.41	12.59	0.29	1.58	12.37				12.48	19.64	0.91
Phenolic acids					16.55	4.88	1.03		14.8				9.84	9.84	16.55
Resveratrol					0.21	0.007	0.007		0.03				0.019	0.02	0.21
Total phenolics ^a^										260	298	228.3	260	262.10	

^1–9^ Citations: 1, USDA Database for the Flavonoid Content of Selected Foods, Release 3.1 [12]; 2, USDA Database for the Proanthocyanidin Content of Selected Foods [14]; 3, Phenol-Explorer [20]; 4, Mullen *et al.* [11]; 5, Dani *et al.* [21]; 6, Stalmach *et al.* [8]; 7, Seeram *et al.* [22]; 8, Bolling *et al.* [23]; 9, Burin *et al.* [24]; ^a^ Total phenolics by Folin-Ciocalteu reaction; ^b^ includes flavanol monomers; ^c^ Average of 7 purple grape juices; * Values presented in the USDA databases are converted from mg/100 g to mg/100 mL by using a factor of 1.0624 g/mL.

### 2.3. Actions of Grape Juice Polyphenols: Dose-Response Relationships

Numerous basic and clinical research investigations have demonstrated that the polyphenols found in grape juice possess bioactivity relevant to the pathogenesis of CVD, including: (i) increasing antioxidant defenses, e.g., scavenging reactive oxygen and nitrogen species; chelating redox-active transition minerals; sparing or synergizing with other antioxidants; modulating redox-sensitive transcription factors; inducing antioxidant enzymes/proteins; inhibiting pro-oxidant enzymes; (ii) reducing the growth of an atherosclerotic plaque, e.g., reducing adhesion molecule express; limiting inflammatory processes; increasing the resistance of LDL to oxidation; (iii) inhibiting platelet aggregation; (iv) enhancing blood pressure regulation and vascular reactivity; and (v) reducing serum cholesterol and triglycerides [7,25,26,27]. 

#### 2.3.1. *In Vitro* and *ex Vivo* Studies

Fitzpatrick *et al.* [28] first tested logarithmic dilutions of Concord grape juice in intact rat aortic rings *in vitro* and found a dose-dependent vasorelaxation action mediated by the cyclic GMP-nitric oxide pathway. Using porcine coronary artery rings and endothelial cells *in vitro*, Anselm *et al.* [29] found Concord grape juice induced endothelium-dependent relaxation of coronary arteries via a redox-sensitive Src- and PI3-kinase/Akt-dependent activation of endothelial nitric oxide synthase (eNOS) and, to a minor extent, of endothelium-derived hyperpolarizing factor (EDHF) in a dose-dependent manner over a range of 0.1–100 mg/L. Similarly, *in vitro*, Concord grape juice was found to inhibit Cu^2+^-induced oxidation of human plasma and of human LDL + VLDL in a dose-dependent fashion from 0.5 to 8 µM and 50 to 100 µM, respectively [30]. The consistency of a dose-responsive relationship for the bioactivity of grape polyphenols is supported by experiments conducted by Liu *et al.* [31] on their antiangiogenic effects in human umbilical vein endothelial cells inhibiting capillary tube formation and matrix metalloproteinase-2 expression.

#### 2.3.2. Animal Models

In hypercholesterolemic hamsters, Vinson *et al.* [32] fed half-strength Concord grape juice to for 10 weeks and, compared to the control group, found it lowered plasma cholesterol, triglycerides, and lipid peroxides as well as the percent atherosclerosis. Similarly, in hypercholesterolemic rabbits, Shanmuganayagam *et al.* [33] fed Concord grape juice at 225 mL/day for 48 days and, compared to the control group, found it attenuated platelet aggregation, reduced serum cholesterol, lowered blood pressure, and slowed atheroma development. However, these studies employed only a single dose, so the dose-response nature of these actions cannot be assessed.

#### 2.3.3. Prospective Cohort Studies

Many prospective observational studies have found an inverse association between consumption of polyphenol-rich foods or individual polyphenols and CVD risk (e.g., see [34,35,36,37]). These data consistently indicate a temporal and dose-response relationship exists between the intake of flavonoids and related polyphenols and risk of CVD. This relationship would similarly be expected to apply to consumption of grape juice across a range of usual intakes or its contribution to total polyphenol consumption when added to usual current intakes. Thus, consideration can be given to the flavonoid and total polyphenol intake obtained from Concord and other purple grape juice (Table 1) with regard to its potential contribution to increasing total consumption to that associated with lower risk of CVD and/or biomarkers of specific risk factors such as vascular adhesion molecules and cytokines.

Employing a sub-cohort of 2115 participants from the Nurses’ Health Study, Landberg *et al.* [38] found total flavonoid consumption was inversely associated with the pro-inflammatory cytokine interleukin (IL)-18 in plasma across quintiles with median intakes at 106.5, 174.1, 242.7, 374.9, and 801.0 mg/day, respectively (*p* = 0.034), in a non-prospective, nested case-control design. Thus, in the context of this dose-response relationship, intakes of 52.34 to 105.13 mg/day of total flavonoids from servings of 118 to 237 mL Concord grape juice could make a substantial contribution by shifting an individual into a higher quintile category. An inverse association (*p* = 0.012) with plasma soluble vascular cell adhesion molecule (sVCAM)-1, a biomarker of endothelial dysfunction and putative pathogenic precursor of CVD, was also observed for the intake of flavonols across quintiles with median intakes at 7.4, 11.0, 14.8, 20.7, and 35.4 mg/day. A similar inverse association, albeit with borderline statistical significance (*p* = 0.09), was observed between the intake of flavonols and IL-18. In the context of this relationship, intakes of 4.29 to 8.59 mg/day of flavonols from servings of 118 to 237 mL Concord grape juice could make a substantial contribution by shifting an individual into a higher quintile category depending on baseline intake.

Investigating data from the longitudinal Rotterdam Study (*n* = 4807), Geleijnse *et al.* [39] reported an inverse association between flavonol intakes (specifically, the sum of kaempferol, myricetin, and quercetin) and fatal myocardial infarction across tertiles with median intakes at 16.8, 27.5, and 40.0 mg/day, respectively, after 5.6 years of follow-up. Flavonol consumption was marginally related (*p* = 0.07) to the risk of all incident myocardial infarctions with a 12.7% reduction in risk per each 10 mg increase in daily intake. Thus, the addition of 4.28 to 8.59 mg flavonols from servings of 118 to 237 mL Concord grape juice could contribute substantially to increasing daily intake by 10 mg.

Examining the Finnish Mobile Clinic Health Examination Survey (*n* = 10,054), Knekt *et al.* [40] observed that after 28 years of follow-up, mortality from ischemic heart disease was inversely correlated (*p* = 0.02) with intakes of the flavonol quercetin across quartiles with median intakes at 1.5, 2.5, and 3.9 mg for men and 1.8, 2.9, and 4.7 mg for women. In the context of this dose-response relationship, an intake of 1.86 to 3.22 mg quercetin found in 118 and 237 mL Concord grape juice, respectively, could make a marked contribution by shifting an individual into a higher quintile category. 

Hollman *et al.* [41] conducted a meta-analysis of 6 prospective cohort studies involving 111,067 people for 6–28 years and evaluated the association between dietary flavonols and stroke. Individual flavonol intakes ranged from about 0.2 to 125 mg/day and were variously assessed in each study by tertiles, quartiles or quintiles. High *versus* low intakes of flavonols were associated with a statistically significant 20% reduction in nonfatal and fatal stroke, though specific dose-response relationships were not described. 

Using data from the 806 participants in the Zutphen Elderly Study, Arts *et al.* [42] found ischemic heart disease mortality was inversely related (*p* = 0.017) to flavanol intakes across tertiles with median intakes at 25.3, 66.8, and 124.0 mg/day after 10 years of follow-up. After adjustment for flavanol intake and tea consumption, a 7.5 mg increase in flavanol intake from sources other than tea was associated with a tendency for a 20% reduction in ischemic heart disease mortality risk (*p* = 0.114). In the context of this dose-response relationship, even independent of consumption of tea flavanols, an intake of 1.65 to 3.32 mg/day of flavanols from a serving of 118 or 237 mL Concord grape juice, respectively, could contribute importantly to the 7.5 mg intake from foods other than tea and a reduction in the risk of CVD. 

In conducting an observational study among 7172 participants of the PREDIMED trial, over an average of 4.3 years of follow-up, Tresserra-Rimbau *et al.* [43] found a 47% reduction in the risk of CVD associated with total polyphenols when comparing the highest to the lowest quintile of intake (1170 *vs.* 562 mg/day; *p* = 0.04). Similarly, a statistically significant reduction of 60% and 33% in the risk of CVD was correlated with the trend across quintiles with the intake of flavanols (90, 129, 158, 192, and 263 mg/day; *p* = 0.003) and anthocyanins (11.8, 23.6, 32.8, 45.7, and 74.6 mg/day; *p* = 0.05), respectively.

Analyzing combined data after 14 years of following 156,957 participants from the prospective Nurses’ Health Study I and II and the Health Professionals Follow-Up Study, Cassidy *et al.* [44] found those participants in the highest quintiles of anthocyanin intake (with a median of 18, 16.2, and 21.9 mg/day in each cohort, respectively) had a lower risk of hypertension (*p* = 0.03) than those in the lowest quintiles (with a median of 6.7, 8.6, and 11.8 mg/day in each cohort, respectively). In this context, the intake of 31.69 to 63.65 mg anthocyanins from servings of 118 or 237 mL Concord grape juice, respectively, would make a substantial contribution to this reduction of elevated blood pressure (BP). Similarly, in a cross-sectional study of 1898 women from the TwinsUK registry, aged 18–75 years, Jennings *et al.* [45] found that anthocyanin intake (from 8.4 to 23.6 mg/day with a mean of 17.7 ± 14.9 mg/day) was associated with lower central systolic blood pressure (*p* = 0.02) as well as lower mean arterial pressure and pulse wave velocity (each at *p* = 0.04) when comparing the highest to the lowest quintile of intake. 

Using data from 34,489 participants in the Iowa Women’s Health Study, Mink *et al.* [46] reported inverse correlations between intake of anthocyanidins and coronary artery disease (*p* = 0.031) and CVD mortality (*p* = 0.032) with only two intake categories, 0 and a median of 0.2 mg/day (range 0.01–1040 mg/day) after 16 years of follow-up. The intake of either 31.69 or 63.65 mg anthocyanins from a 118 or 237 mL serving of Concord grape juice, would make a substantial contribution to the reduction in risk of CVD in the context of this cohort.

Analyzing data from the prospective Nurses’ Health Study II of 93,600 women, Cassidy *et al.* [47] found that high anthocyanin intake was associated with a reduced risk of myocardial infarction in young and middle-aged women, 25 to 42 years, after 18 years of follow-up. The trend for this relationship was noted across quintile medians of anthocyanin intake at 2.5, 5.0, 8.4, 13.5, and 25.1 mg/day (*p* = 0.047). Daily consumption of Concord grape juice at 118 or 237 mL/day would provide 31.69 or 63.65 mg anthocyanins and readily place a woman within this highest quintile of intake.

McCullough *et al.* [48] reviewed a seven-year follow-up of the Cancer Prevention Study II Nutrition Cohort (*n* = 38,180 men and 60,289 women) with a mean age of 70 and 69 years, respectively. Comparing participants with total flavonoid intakes in the top *versus* bottom quintiles of intake (512.5 and 94.5 mg/day, respectively) had a lower risk of fatal CVD (*p* = 0.01). Interestingly, anthocyanidins, flavanols, flavones, flavonols, and proanthocyanidins were each individually associated with lower risk of fatal CVD. The trend for this relationship was noted across quintile medians of anthocyanidin intake at 3.8, 6.8, 9.8, 13.7, and 22.2 mg/day (*p* = 0.04); of flavanol intake at 7.0, 11.8, 16.8, 26.3, and 63.7 mg/day; of flavonol intake at 6.9, 9.9, 13.0, 17.2, and 27.2 mg/day (*p* = 0.03); and of proanthocyanidin intake at 53.1, 90.0, 132.0, 196.8, and 379.4 mg/day (*p* = 0.02). Importantly, many of the associations reported by these investigators appeared to be nonlinear, with lower risk at intakes above the referent category. Additionally, most inverse associations appeared with intermediate intakes, further suggesting that even relatively small amounts of flavonoid-rich foods may be beneficial. In the context of this cohort, consumption of 52.34 to 105.13 mg total flavonoids, 31.69 to 63.65 mg anthocyanins, 1.65 to 3.32 mg flavanols, 4.28 to 8.59 mg flavonols, and 14.73 to 29.58 mg proanthocyanidins from servings of 118 and 237 mL Concord grape juice, respectively, may contribute to the reduction of fatal CVD.

#### 2.3.4. Clinical Trials

In clinical trials, Concord grape juice and an unspecified purple grape juice have been shown to improve flow-mediated vasodilatation (FMD), platelet function, and platelet-dependent inflammatory responses in patients with hypercholesterolemia or coronary artery disease [30,49,50,51,52,53,54,55,56,57,58,59,60] (Table 2). Over a 56-day duration, at 5.5 mL/kg/day, Concord grape juice reduced BP in moderately hypertensive patients [52] and at 7 mL/kg/day lowered nocturnal BP in pre-hypertensive and Stage 1 hypertension patients [54]. Examining biomarkers of oxidative stress, O’Byrne *et al.* [55] found consumption of 10 mL/kg/day Concord grape juice for 14 days increased both serum total antioxidant activity and the resistance of LDL against oxidation and reduced protein carbonyl concentration in healthy subjects. Similarly, Vinson *et al.* [30] reported an increase in the *ex vivo* resistance of LDL to oxidation following the consumption of 400 mL/day Concord grape juice for seven days. In a randomized clinical trial with a cross-over design in healthy smokers, Siasos *et al.* [56] found that drinking Concord grape juice at 7 mL/kg/day for 14 days improved endothelial function (via assessment of FMD) and vascular elastic properties (via determination of pulse wave velocity) of the arterial tree and attenuated acute smoking-induced impairment of these properties of the arterial wall. Other randomized clinical trials, similarly employing only a single dose of Concord or concentrated red grape juice, have demonstrated a benefit on other biomarkers of oxidative stress and inflammation, including reductions in urinary F_2α_-isoprostane excretion, superoxide production, CD40L release, tumor necrosis factor-α, plasma monocyte chemoattractant protein 1 [49,55,57,58].

**Table 2:** Clinical evidence of grape juice on risk factors of cardiovascular disease.

Study	Study Design	Subjects	Treatment	Duration (Day)	Selected Outcomes Mediated by Grape Juice
Vinson *et al.* [30]	crossover (1 week washout)	6 adults (3M, 3F)	400 mL/day Concord GJ or placebo beverage	7	↑ lag time of LDL oxidation by 27%
Albers *et al.* [49]	crossover (2 weeks washout)	20 adults with coronary heart disease	7/mL/kg/day GJ or calorie matched placebo	14	↓ Souble CD40L by 38% ↔ platelet aggregation, hsCRP, and IL-8
Coimbra *et al.* [50]	Crossover (2 weeks washout)	Hypercholesterolemic patients (8 M, 8 F, 51.6 years)	250 mL/d red wine or 500 mL/day purple GJ	14	↓ ICAM-1 by 21% ↑ FMD by 55% ↔ lipid profile, blood glucose and platelet aggregation
Stein *et al.* [51]	single arm	15 patients with coronary artery disease (12 M, 3 F, 62.5 years)	8 mL/kg/day Concord purple GJ	14	↑ FMD by 191% ↑ lag time of LDL oxidation by 35% ↑ TC, TG, and insulin by 16%, 51%, and 172%
Park *et al.* [52,53]	parallel	40 Korean hypertensive men (44.5 years)	5.5 mL/kg/day Concord GJ or calorie matched placebo	56	↓ SBP and DBP by 7.2 and 6.2 mmHg ↔ lipid profile ↔ DNA damage in lymphocytes
Dohadwala *et al.* [54]	crossover (4 weeks washout)	64 patients with prehypertension and stage I hypertension (44 M, 20 F, 42.5 years)	7/mL/kg/day Concord GJ or calorie matched placebo	56	↓ blood glucose by 2% ↔ lipid profile
O’Byrne *et al.* [55]	parallel	32 healthy adults (13 M, 19 F, 28 years)	400 IU/d RRR-α-tocopherol or 10 mL/kg/day Concord GJ	14	↓ plasma protein carbonyl by 20% ↑ lag time of LDL oxidation by 10% ↑ TG by 42% ↔ TC
Siaosos *et al.* (56)	parallel	26 healthy smokers (10 M, 16 F, 26 years)	7/mL/kg/day 100% Concord GJ or grapefruit juice	14	↑ FMD by 13.7% ↔ lipid profile, blood glucose ↔ BP
Castilla *et al.* [57]	parallel	32 hemodialysis patients (16 M, 16 F, 33–79 years)	100 mL/day concentrated red GJ, 800 IU/d vitamin E, both or placebo	14	↓ LDL-C by 17% ↑ HDL-C by 22.5% ↓ oxidized LDL by 66% ↓ MCP-1 by 3.7% ↔ VCAM-1, ICAM-1, and hsCRP
Freedman *et al.* [58]	single arm	20 healthy adults (12 M, 8 F, 30.6 years)	7/mL/kg/day GJ	14	↓ platelet aggregation by 33.2% ↑ platelet NO production by 71%
Chou *et al.* [59]	parallel	22 patients with coronary artery disease (18 M, 4 F, 64 years)	4 or 8 mL/kg/day Concord purple GJ and then GJ plus 400 IU vitamin E	56 (GJ) and 28 (GJ + vit E)	↑ FMD by 167 and 154% by GJ and vit E did not improve further ↔ lipid profile, glucose, and insulin
Keevil *et al.* [60]	crossover (1 week washout)	10 healthy adults (5 M, 5 F, 42 years)	5–7.5 mL/kg/day 100% purple GJ or grapefruit juice	7–10	↓ platelet aggregation by 77%

Chou *et al.* [59] tested two doses of Concord grape juice, 4.6 ± 0.3 and 8.0 ± 0.6 mL/kg (each with *n* = 11), divided into two daily servings in patients with coronary heart disease and found the improvement in FMD was similar between two groups. These results suggest a possible upper threshold effect for the benefit of Concord grape juice on FMD. Alternatively, the polyphenol dose in this study, especially the concentration of anthocyanins in the high dose treatment, may have been markedly lower than intended as the juice had been stored for 4–6 months without refrigeration. An earlier experiment had shown a reduction of the flavonoid content of Concord grape juice after testing at extreme storage conditions (35 °C for 18 months in R-enamel cans) [61] but its direct relevance to the results of Chou *et al.* [59] are not clear.

These and additional protocols, employing only a single dose of grape juice and within a relatively narrow range of intake make it difficult to discern whether there is a clear relationship between the intake of polyphenols and the measured outcome variable. However, combining these data with well-designed randomized clinical trials of other polyphenol-rich foods and beverages describing the same outcomes but covering a broader range of intake can help establish a perspective in which to substantiate dose-response relationships for 100% Concord or purple grape juice.

## 3. Results: Polyphenol Dose-Response Relationships

### 3.1. Brachial Artery Flow-Mediated Dilation

**Figure 1:**
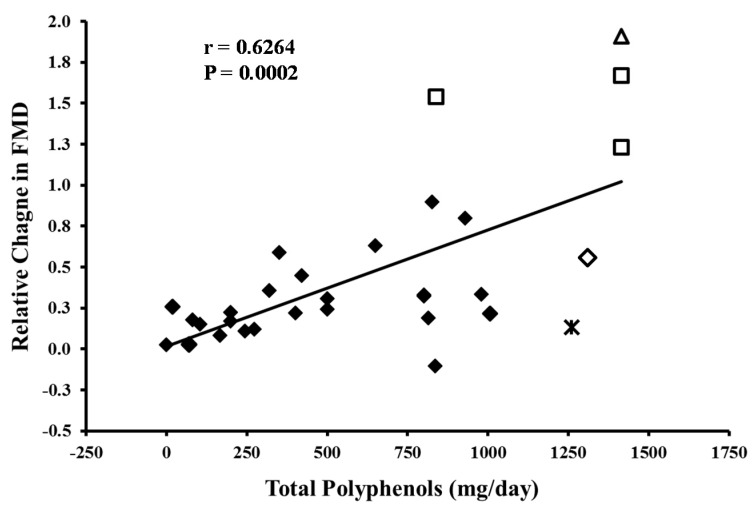
Polyphenol-FMD dose-response for polyphenol-containing foods and beverages. The relative change in FMD for each study was calculated as: (Follow-up FMD − Baseline FMD)/Baseline FMD. Regression equation: Relative Change in FMD = (Total polyphenols/d × 0.00071) + 0.0187. Concord grape juice: 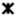 Siaosos *et al.* [56] 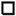 Chou *et al.* [59]; 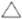 Stein *et al.* [51]; Other purple grape juice: 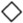 Coimbra *et al.* [50]; Other flavonoid-containing foods and beverages: ♦.

Clinical studies of Concord grape juice have used doses of 355 to 710 mL, volumes larger than generally consumed at one time. We addressed the question of whether the available evidence supports the contention that lower amounts of Concord grape juice would be expected to have favorable effects on the cardiovascular system. We plotted the relative change in FMD *versus* daily polyphenol dose from 16 chronic studies of other beverages and foods and from 4 studies of purple grape juice. To adjust for differences in FMD methodology, the relative change in FMD for each study was calculated as: (Follow-up FMD—Baseline FMD)/Baseline FMD. The relative change expression is employed instead of the relative percent change because a value of the former obtained from the regression equation presented in Figure 1 can be readily used to generate the corresponding FMD change mediated by a given polyphenol content. Data from the placebo group of studies including such an arm were not included in the analyses as such adjustments for some studies but not others could confound the analyses. It is useful to note that with this approach of using the relative change value, the clinical significance (but not necessarily the statistical significance) of polyphenol-rich foods or beverages on FMD improvement may be masked. For example, the baseline FMD for healthy individuals and smokers is often about 9% and 2%, respectively. An increase in FMD after consumption of grape juice or other polyphenol rich food by 1% would double the value of the relative change in smoker but be comparatively small in healthy individuals. The non-grape juice studies were selected based on two specific criteria: (i) the availability of information about the polyphenol content of the studied intervention and (ii) the inclusion of one or more of four CVD risk factors, *i.e.*, FMD, BP, platelet aggregation, and the resistance of LDL to oxidation. The selected articles included studies of chokeberry juice [62], black tea [63], berry anthocyanins [64], anthocyanin-rich blackcurrant juice [65], citrus flavonoids [66], boysenberry [67], flavonol-rich cocoa [68,69,70,71], dark chocolate [72,73,74], and the control interventions. For purple grape juice, we included the 3 studies of Concord grape juice [51,56,59] and a study of a purple grape juice of unspecified source [50]. 

The studies were selected after the PubMed search using the keywords, polyphenol, flow mediated dilation or FMD, blood pressure, platelet, LDL oxidation, and chronic clinical trial. In all 4 figures the dose of administered polyphenols in the cited studies are expressed based on either principle flavonoid, all quantified flavonoids or total phenols as gallic acid equivalents (GAE) determined by the Folin Ciocalteu assay. Among the 15 studies reviewed for the impact of polyphenol on FMD, eight trials used the principle flavonoid or sum of all quantified flavonoids [62,64,66,68,69,70,71,73], six used a Folin value [51,56,59,63,65,67], and one was not specified [50]. The studies enrolled healthy individuals and patients with mild hypercholesterolemia, cigarette smokers, diabetes mellitus, and coronary artery disease. The age of the subjects ranged from 26 to 64 years and the treatment period ranged from one to eight weeks. Despite the variability in subject characteristics and treatment time, there was a highly significant correlation between daily polyphenol dose and relative change in FMD (Figure 1). 

The open triangle, open squares, and star in Figure 1 display the responses from the different subgroups enrolled in the Concord grape juice studies by Stein *et al.* [51], Chou *et al.* [59], and Siaosos *et al.* [56], respectively. Since the body weight of the participants was not reported in those studies, we calculated the daily polyphenol intake using an average weight of 80 kg as indicated by Stein *et al.* [51] and Chou *et al.* [59] (from Table 1). Stein *et al.* [51] reported an improvement in FMD from 2.2% to 6.4% (relative change = 1.91) over two weeks with 8 mL/kg/day (1414 mg/day). Chou *et al.* [59] reported an improvement from 1.3% to 3.3% (relative change = 1.54) following consumption of 8 mL/kg/day and an improvement from 1.2% to 3.2% (relative change 1.67) following consumption of 4 mL/kg/day for eight weeks. They also reported an improvement from 1.3% to 2.9% (relative change = 1.23) after four weeks of treatment. Siaosos *et al.* [56] reported an average improvement of 1.44% (relative change = 0.14) following consumption of 7 mL/kg/day (1259 mg polyphenols/day) for two weeks in otherwise healthy smokers. The study by Coimbra *et al.* [50] involved consumption of 500 mL/day purple grape juice in a group of patients with hypercholesterolemia. They observed an increase in FMD from 10.9% to 16.9% (relative change = 0.55). Assuming this juice contained 1310 mg GAE polyphenols/L, we plotted this response as the open diamond, and found it to be close to the predicted value based on regression analysis.

Overall, there appears to be a strong relationship between daily total polyphenol dose and change in FMD observed in chronic beverage intervention studies. Although the administered amounts in the 100% Concord grape juice studies represent the upper extreme in regard to daily polyphenol intake, this analysis provides good evidence that lower amounts of Concord grape juice would have clinically significant effects on FMD. For example, based on regression analysis (relative change in FMD = (Total polyphenols/d × 0.00071) + 0.0187) presented in Figure 1, consumption of 118 and 237 mL of Concord grape juice daily, containing 307.6 and 615.2 mg polyphenols would be expected to produce a relative improvement in FMD of 0.24 (e.g., FMD of 5.00% to 6.19%) and 0.46 (e.g., FMD of 5.0% to 7.27%), respectively. Interestingly, such improvements in FMD are comparable to the relative improvement of 0.32 observed following weight loss [75] and of 0.16 observed following smoking cessation [76]. Such interventions are known to markedly reduce the risk for cardiovascular events, suggesting that the effects of purple grape juice on FMD are clinically relevant. It is noteworthy that the studies used in this analysis included healthy individuals as well as patients with cardiovascular disease. Thus, it is reasonable to conclude that these clinically significant effects would extend to the general population.

### 3.2. Blood Pressure

**Figure 2:**
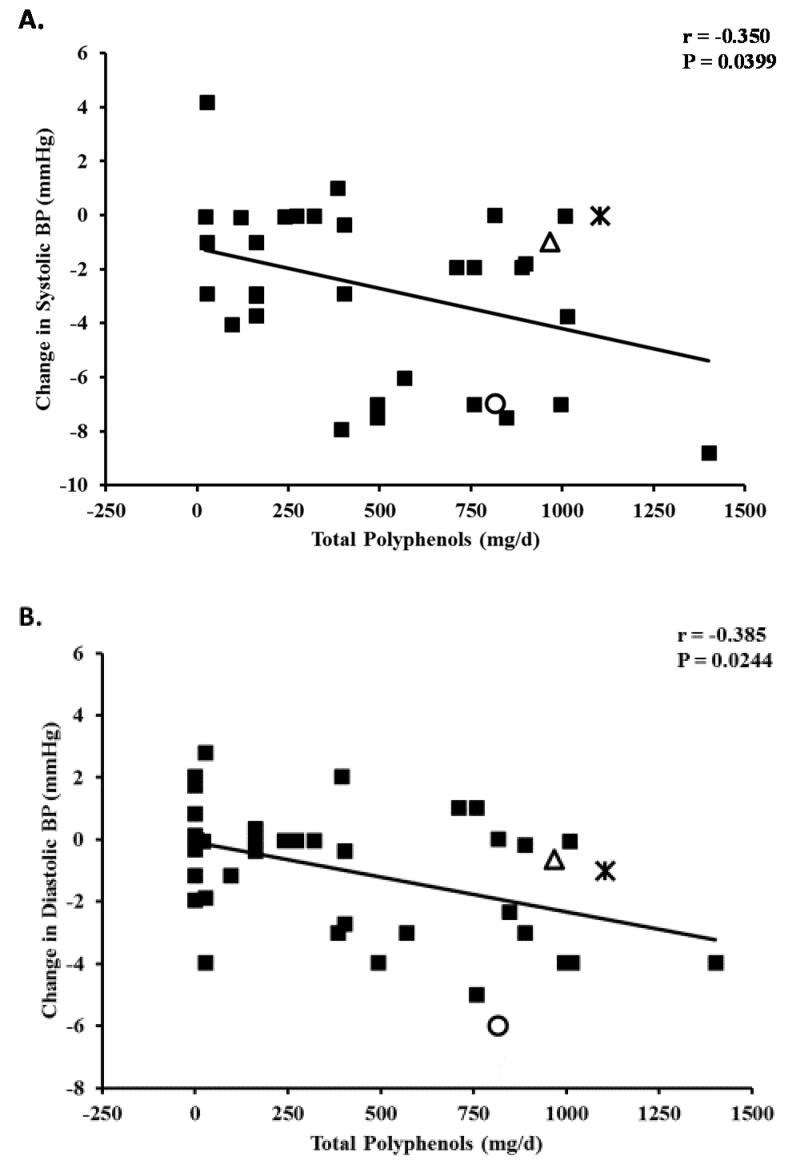
Dose-response for polyphenol-containing foods and beverages on systolic blood pressure (**A**) and diastolic blood pressure (**B**). Regression equations: Change in Systolic BP = (Total polyphenols/day × −0.00294) − 1.2585. Change in Diastolic BP = (Total polyphenols/day × −0.00205) − 0.2279. Concord grape juice: 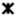 Siaosos *et al.* [56] 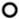 Park *et al.* [52]; 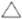 Dohadwala *et al.* [54].

We completed a similar analysis to that of FMD for systolic BP (SBP) and diastolic BP (DBP). We selected clinical trials that examined the BP effects of foods and beverages (blackcurrant juice, dark chocolate, cocoa, green and black tea) and polyphenol supplements (grape seed extract, berry anthocyanins, citrus polyphenols, quercetin, epigallocatechin gallate [EGCG]) in healthy subjects and in subjects with hypertension, pre-hypertension, obesity, and the metabolic syndrome [64,65,66,72,77,78,79,80,81,82,83,84,85,86,87,88,89,90]. We also included studies of the effect of Concord grape juice on BP in patients with hypertension [52,53] and healthy subjects with high normal BP [54]. We found a highly significant relationship between total daily polyphenol dose expressed as either GAE [52,53,54,65,72,77,78,84,90] or principle flavonoids [64,66,79,80,81,82,83,85,86,87,88,89] and decreases in SBP and DBP (Figure 2). However, there were lower correlation coefficient values for the BP studies compared to the FMD studies. This finding likely reflects greater heterogeneity in the supplements administered, subject population, and duration of treatment as well as more complex mechanisms for BP regulation. The effect of Concord grape juice on BP in the protocol by Park *et al.* [52] is close to the predicted effect. In the trial by Dohadwala *et al.* [54], the observed 1 mmHg reduction in SBP and DBP were not statistically significant, but were close to the predicted values.

These findings suggest that serving sizes of 118 and 237 mL for Concord grape juice would be expected to have clinically significant effects on BP. Based on the regression analysis (change in SBP = (Total polyphenols/day × −0.00294) − 1.2585 and change in DBP = (Total polyphenols/day × −0.00205) − 0.2279), these two serving sizes of 100% Concord grape juice daily containing 307.60 and 615.20 mg polyphenols would be predicted to produce a reduction in SBP of 2.16 and 3.07 mmHg and in DBP of 0.86 and 1.49 mmHg, respectively. If sustained over a period of years, such reductions in BP would be expected to have important beneficial effects on cardiovascular outcomes [91].

### 3.3. Platelet Aggregation 

**Figure 3:**
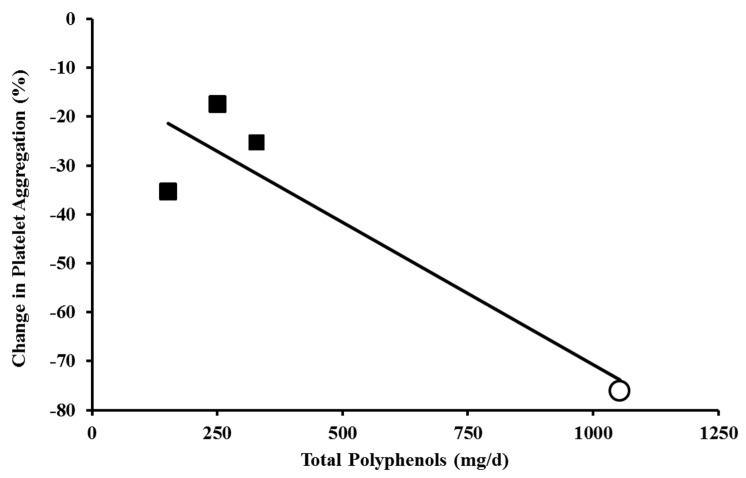
Relationship between the intake of polyphenol-containing foods and *ex vivo* collagen-induced platelet aggregation. Concord grape juice: 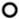 Keevil *et al.* [60].

Two prior studies examined the effects of Concord grape juice on platelet aggregation. Keevil *et al.* [60] observed a 77% reduction in collagen-induced platelet aggregation using platelet aggregometry following Concord grape juice consumption in healthy volunteers. Freedman *et al.* [58] showed 34% reduction from 57.6% to 38.6% in phorbol ester-induced platelet aggregation following Concord grape juice intake. They also observed reduced production of reactive oxygen species and increased production of nitric oxide following Concord grape juice consumption. It is difficult to compare these studies of grape polyphenol intake and platelet function because of the marked differences in methodology. Two prior studies examined *ex vivo* platelet aggregation in response to a comparable concentration of collagen that was used by Keevil *et al.* [60]. One study examined the effects of two doses of quercetin [92] and the other examined collagen-induced platelet aggregation after consumption of cocoa and placebo. The relation between total polyphenol intake and collagen-induced platelet aggregation in these three studies is shown in Figure 3. While this sample size is small, there is a suggestion of a dose-relationship. In a review of dietary polyphenols and platelet function, Ostertag *et al.* [93] suggested that there are dose-dependent effects of polyphenol-containing foods and beverages on platelet function using different methodology. As with measures of vascular reactivity and BP, the available evidence indicates a dose-dependent effect of polyphenol-containing foods on platelet aggregation, and supports the contention that standard serving sizes of purple grape juice daily could have clinically meaningful effects on platelet function in the general population.

### 3.4. Resistance of LDL to Oxidation

**Figure 4:**
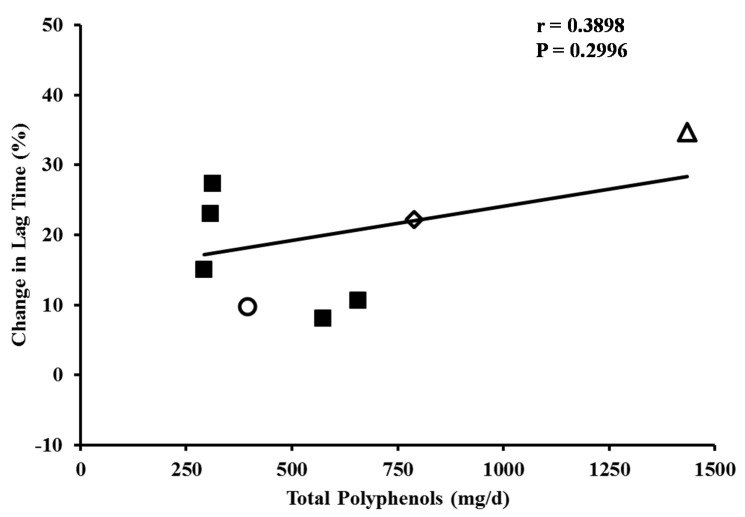
Dose-response of LDL lag time for polyphenol-containing foods and beverages. Regression equations: Change in lag time = (Total polyphenols/d × 0.00974) + 14.3443. Concord grape juice: 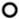 O’Byrne *et al.* [55]; 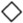 Vinson *et al.* [30]; 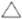 Stein *et al.* [51].

Three prior studies examined the effects of Concord grape juice on the susceptibility of LDL to Cu^2+^-mediated oxidation as assessed by lag time prior to initiation of conjugated diene formation. Stein *et al.* [51] observed a 34% increase in lag time following consumption of Concord grape juice in patients with coronary artery disease. In healthy volunteers, Vinson *et al.* [30] observed a 22% increase in lag time and O’Byrne *et al.* [58] found a 10% increase in lag time with Concord grape juice. Several other studies examined the lag time prior to Cu^2+^-mediated LDL oxidation following consumption of other foods and beverages, including wine polyphenols, cocoa, chocolate, green and black tea, and the corresponding control foods and beverages [94,95,96,97,98]. Among the reviewed studies, total polyphenols were expressed as GAE in five trials [30,51,58,97,98] and as principle flavonoids in the remaining three studies [94,95,96]. The relation between total polyphenol intake and change in lag time is plotted in Figure 4 and reveals a strong dose-relationship. Thus, the consumption of polyphenol-containing foods and beverages can increase the resistance of LDL to *ex vivo* oxidation. This finding is consistent in both healthy individuals and in patients with coronary artery disease. Based on regression analysis, consumption of 118 or 237 mL of 100% Concord grape juice daily containing 307.6 and 615.2 mg polyphenols would be predicted to produce a 16% and 19% increase in lag time, respectively. However, the lack of a significant correlation between the lag time and total polyphenol diminishes the accuracy of this prediction. 

## 4. Discussion

Ruxton *et al.* [99] have suggested there is no evidence that 100% fruit juices are any less beneficial in reducing the risk of CVD than from their respective whole fruits. They note that despite the lack of fiber, pure fruit juices “do appear to possess the necessary nutrients for CVD risk reduction”, noting particularly their polyphenol content. Similarly, in their review, Clemens *et al.* [3] conclude that 100% fruit juice in appropriate amounts help individuals to meet fruit recommendations and benefits without any untoward impact on energy intake or food costs. However, neither of these reports examined the specific dose-response relationships between polyphenol intake and CVD outcomes. In addition to the evaluative process outlined by Lupton *et al.* [4] for dietary bioactive components, the classic work of Sir Austin Bradford Hill stresses the importance of a “biological gradient or dose-response curve” in distinguishing between association and causation [100]. Thus, with a very limited number of direct studies examining the dose-response relationship between pure purple grape juice and physiological responses relevant to CVD risk, we placed the available information, mostly from studies of Concord grape juice, within the context of published results from clinical trials on other flavonoid-rich foods, focusing particularly on those using essentially the same methods of assessment and using serving sizes typically consumed. With regard to fruit juice, it is worth noting that the 2010 Dietary Guidelines for Americans and the Dietary Approaches to Stop Hypertension diet [101] equate one cup of fresh fruit with one cup of 100% fruit juice and recommend 1–2 and 2.0–2.5 cup-equivalents per day, respectively. While 0.5 to 1 cup (4 to 8 oz or 118 to 237 mL) of 100% Concord grape juice provides 306.8 to 616.2 mg polyphenols, the dose-response relationships quantified here suggest that lower intakes, e.g., 0.5 cup (4 oz), can provide sufficient intakes of these bioactives to affect the CVD risk factors like BP, FMD, platelet aggregation, and LDL oxidation. 

There are several limitations associated with our approach to combine the results of studies of different polyphenol-rich foods to assess whether a dose-response relationship exists such that results from limited studies of one beverage (Concord grape juice) can be assessed within the context of several other foods and beverages. One limitation is the common use in many studies of different analytical methods with varying degrees of accuracy and precision for the determination and expression of the polyphenol content of the foods, e.g., via the principle flavonoid, all quantified flavonoids or total phenols as gallic acid equivalents determined by the Folin Ciocalteu assay. As these different values cannot be standardized to a common denominator, we treated each as being essentially equivalent to the others. Another limitation to our approach is the reliance on studies not only of different durations in different populations but of different designs. For example, 25 of the studies used in our comparisons employed a parallel or crossover design with a control (placebo) and treatment group [30,49,52,53,63,64,65,66,71,72,73,74,77,78,79,80,81,82,83,84,85,86,87,88,89] while 21 others tested treatment arm(s) with measurements taken at baseline and the end of the trial [50,51,54,55,56,57,58,59,60,62,67,68,69,70,71,72,73,74,92,94,95]. We included both approaches and utilized only the treatment arm in our analysis so as to analyze all studies similarly. It is not uncommon for studies of whole foods to not include a control arm in part because it is not always clear what might comprise an appropriate placebo for a whole food; indeed, different studies use different types of dietary controls, e.g., matched for calories, macro- or micronutrient composition, volume, low *vs.* high phytochemical content, *etc*. 

Our regression analysis of the association between total polyphenol intake and these four risk factors reveals that the biological effect of polyphenols appears not to have reached a maximum efficacy as there is no sign of saturation on the biological responses. These results echo a common call for larger and longer randomized clinical trials designed to provide more definitive evidence of health benefits from polyphenol-rich foods and beverages. If this goal is to be met, then this approach to dose-response analysis may be useful for determining the serving size to be chosen for these protocols. However, it is worthwhile to note there are some limitations to this approach. For example, we combined studies in which the characteristics of the study subjects differed markedly, e.g., healthy adults as well as those who were smokers or had hypercholesterolemia, hypertension or diabetes. Further, there was a wide range in the duration of the interventions, e.g., from one to eight weeks, though none were truly long-term trials. While a differential efficacy of the several classes of polyphenols would be anticipated, this parameter was not captured in our analysis. Nonetheless, despite these differences in the subjects and trial duration, significant correlations were observed between the daily polyphenol dose and FMD. Indeed, the consistency of dose-response relationship across such heterogeneous groups suggests this beneficial relationship will extend to the general population. Inverse dose-response relationships, albeit with a lower correlation coefficient, were noted as well for polyphenol intakes and both SBP and DBP. This weaker relationship may result from BP outcomes being more sensitive to variation among the study subjects than FMD. Correlations between a wide dose range of polyphenols and the resistance of LDL to oxidation were also weak, probably due to a small sample size in the analysis. While the number of available studies and their respective sample sizes were quiet small, the regression analysis suggests a dose-response relationship as well for polyphenols and platelet aggregation. While the limitations with using intermediary biomarkers of CVD are widely appreciated, the ability to assess together an array of indices like those examined here provides some confidence about our ability to recommend flavonoid-rich whole foods and pure fruit juices. Nonetheless, future research examining specific flavonoids or flavonoid profiles of individual foods and juices is warranted as it may uncover significant differences in their impact on these biomarkers. 

Consistent with results of these regression analyses, McCullough *et al.* [48] noted that benefits of flavonoid consumption could be realized at relatively low intake thresholds. For example, with the example of 100% Concord grape juice, consumption of either 0.5 or 1.0 cup would provide a meaningful dose of flavonoids as extrapolated from both prospective cohort studies and clinical trials. Replicating and expanding upon these dose-response relationships should allow for the establishment of more quantitative dietary guidelines for food sources rich in specific flavonoids for CVD risk reduction. This approach could provide useful references for intake before results from long-term randomized clinical trials are available. 

## 5. Conclusions

We have reviewed the available evidence from human studies supporting a role of Concord grape juice polyphenols in CVD risk reduction via consideration of their quantitative contribution to total dietary intake from observational research examining CVD endpoints and dose-response relationships on intermediary biomarkers determined in clinical trials. A similar approach is warranted to support the accumulating evidence suggesting that consumption of purple grape juice can positively influence risk factors associated with cancer, diabetes, impaired immune responsiveness, age-associated cognitive declines, and neurodegenerative diseases [102]. 
